# The Clinical Influence after Implementation of Convolutional Neural Network-Based Software for Diabetic Retinopathy Detection in the Primary Care Setting

**DOI:** 10.3390/life11030200

**Published:** 2021-03-05

**Authors:** Yu-Hsuan Li, Wayne Huey-Herng Sheu, Chien-Chih Chou, Chun-Hsien Lin, Yuan-Shao Cheng, Chun-Yuan Wang, Chieh Liang Wu, I.-Te Lee

**Affiliations:** 1 Division of Endocrinology and Metabolism, Department of Internal Medicine, Taichung Veterans General Hospital, Taichung 40705, Taiwan; brightlight720720@gmail.com (Y.-H.L.); whhsheu@vghtc.gov.tw (W.H.-H.S.); 2 Department of Computer Science & Information Engineering, National Taiwan University, Taipei 10617, Taiwan; 3 School of Medicine, National Yang-Ming University, Taipei 11221, Taiwan; 4 Rong Hsing Research Center for Translational Medicine, National Chung Hsing University, Taichung 40227, Taiwan; 5 Department of Ophthalmology, Taichung Veterans General Hospital, Taichung 40705, Taiwan; doctorccc@gmail.com (C.-C.C.); royalmaple9006@yahoo.com.tw (C.-H.L.); elock816@gmail.com (Y.-S.C.); cywang@vghtc.gov.tw (C.-Y.W.); 6 Department of Critical Care Medicine, Taichung Veterans General Hospital, Taichung 40705, Taiwan; clwu@vghtc.gov.tw; 7 Department of Automatic Control Engineering, Feng Chia University, Taichung 40703, Taiwan; 8 School of Medicine, Chung Shan Medical University, Taichung 40201, Taiwan; 9 College of Science, Tunghai University, Taichung 40704, Taiwan

**Keywords:** area under the curve, diabetes, deep learning, image, retinopathy

## Abstract

Deep learning-based software is developed to assist physicians in terms of diagnosis; however, its clinical application is still under investigation. We integrated deep-learning-based software for diabetic retinopathy (DR) grading into the clinical workflow of an endocrinology department where endocrinologists grade for retinal images and evaluated the influence of its implementation. A total of 1432 images from 716 patients and 1400 images from 700 patients were collected before and after implementation, respectively. Using the grading by ophthalmologists as the reference standard, the sensitivity, specificity, and area under the receiver operating characteristic curve (AUC) to detect referable DR (RDR) were 0.91 (0.87–0.96), 0.90 (0.87–0.92), and 0.90 (0.87–0.93) at the image level; and 0.91 (0.81–0.97), 0.84 (0.80–0.87), and 0.87 (0.83–0.91) at the patient level. The monthly RDR rate dropped from 55.1% to 43.0% after implementation. The monthly percentage of finishing grading within the allotted time increased from 66.8% to 77.6%. There was a wide range of agreement values between the software and endocrinologists after implementation (kappa values of 0.17–0.65). In conclusion, we observed the clinical influence of deep-learning-based software on graders without the retinal subspecialty. However, the validation using images from local datasets is recommended before clinical implementation.

## 1. Introduction

Diabetic retinopathy (DR) is the leading cause of blindness among working-age patients with type 2 diabetes [1]. The prevalence of DR is approximately 24–35% for patients with type 2 diabetes worldwide [2,3,4,5,6], and the burden of vision-threatening DR has been increasing owing to the rapid growth of the diabetic population [5,6,7]. Previous studies have shown that early screening and timely treatment can reduce the risk of worsening DR and blindness [8], and international guidelines recommend that screening for DR should be performed at least once every year for patients with type 2 diabetes [9]; however, adherence to this DR screening program has been alarmingly low [2]. The major barrier to annual DR screening is the lack of trained specialists and equipment to handle the rapidly growing population of patients with diabetes [10].

The use of deep-learning algorithms in the field of DR screening has demonstrated promising results [11]. Recent publications have shown that the diagnostic performance of some deep learning algorithms is similar to, or even better than, that of human experts [12,13]. In addition, these algorithms have the advantage of high reproducibility and they could help to reduce time and human resources theoretically. However, how to translate these advantages into clinical benefits is still in the center of discussion [14,15]. Currently, most studies discussing the use of deep learning algorithms for DR screening have focused on comparing the performance of the algorithms with diagnosis by local retinal specialists or regional graders [16,17]. The change in the clinical workflow or the impact on regional graders after implementation of such software has seldom been discussed.

VeriSee^TM^ (Acer Inc., New Taipei City, Taiwan), a deep-learning-based software for DR, has recently been approved by the Taiwan Food and Drug Administration as a smart medical device based on its comparable performance to that of ophthalmologists [18]. In this study, we deployed VeriSee^TM^, referred to here as “the software”, in an endocrinology department. We compared the diagnostic accuracy of referable DR (RDR) between regional graders and the software before its implementation and investigated the change in the clinical workflow and the influence on regional graders after its implementation.

## 2. Materials and Methods

### 2.1. Setting and Participants

This cross-sectional study was conducted at Taichung Veterans General Hospital between June and October 2019. The payment for performance program for diabetes in Taiwan recommends that patients with diabetes should receive annual comprehensive screening for diabetic complications, including DR [19]. We included patients with type 2 diabetes who underwent a fundus examination during the study period in our hospital.

### 2.2. Retinal Imaging

The standard protocol was performed in a dark room to ensure the physical dilation of the pupils. Retinal images were captured in a single-field, 45-degree view by trained technicians using a digital retinal camera (CR-2, Cannon Inc., Tokyo, Japan). Images were retaken if the technician considered them to be of poor quality, and only the image with the best quality from the repeated assessments was uploaded. All images were collected anonymously for analysis. Finally, adequate image quality was judged independently by each grader. The criterion for excluding an image was a poor quality, as judged by any one of the graders. At the patient level, a patient was excluded if an image of either eye was of poor quality.

### 2.3. Reference Grading

Three ophthalmologists independently graded all retinal images according to the international clinical classification of the DR scale, which classifies DR into no DR, mild non-proliferative DR (NPDR), moderate NPDR, severe NPDR, or proliferative DR (PDR). Moderate NPDR, severe NPDR, and PDR are all identified as RDR. The ophthalmologists graded the images independently and blindly from the output of the software. Disagreements between two ophthalmologists were adjudicated by the senior retinal specialist. Only images that were graded the same by at least two ophthalmologists were included in the final analysis, and the grades served as the ground truth.

### 2.4. The Deep Learning Algorithm

The development of the software has been described previously [18]. Briefly, the model was built by the convolutional network. The base structure for the convolutional network model is Inception V4. The number of layers, neurons, loss function, and active function are the same as inception V4, and other hyperparameters were fine-tuned to obtain the optimal accuracy [20]. After pretraining with large public retinal image datasets, the deep learning model was fine-tuned with 5649 retinal images. The final model was found to detect RDR with a sensitivity of 89.2%, a specificity of 90.1%, and an area under the receiver operating characteristic curve (AUC) of 0.950 [18].

### 2.5. Clinical Workflow

In our clinical practice, retinal images were graded by five endocrinologists. Generally, approximately 700 patients received fundus examinations per month, and five endocrinologists were responsible for all of them. Each endocrinologist was assigned to retinal images from approximately 140 patients and was requested to complete grading of all images within three days after the examination. After software was integrated into our clinical workflow, the preliminary VeriSee^TM^ report accompanied with corresponding retinal images was automatically uploaded in the reports system, which endocrinologists could read before making the final decision in October 2019. Under the awareness of the diagnostic accuracy of the VeriSee^TM^, endocrinologists could either confirm the grading if they agreed with the preliminary report or revise the grading if they disagreed with it after examining the image (Supplementary Figures S1 and S2).

### 2.6. Statistical Analysis

The prevalence of DR is approximately 27–35% in Taiwan [21,22]. With a predefined sensitivity of 86%, a type I error of 5%, a power of 80%, and a margin of error of 7%, the sample size was estimated to be at least 350 patients. To assess the performance of the software and the endocrinologists, the sensitivity, specificity, F1 score, balanced accuracy, and AUC for detecting RDR were calculated using the grading by the ophthalmologists as the ground truth. Different from the software which generates the diagnosis of RDR based on each image, regional graders diagnose RDR at the patient level, which means clinicians would consider referring a patient to an ophthalmologist if one of the patient’s eyes was diagnosed with RDR. To address the gap between the laboratory and the real world, we evaluated the performance of this software at both the patient level and the image level and compared the performance to the regional grader at the patient level. A 95% confidence interval (CI) was obtained based on the exact binominal distribution.

To evaluate the influence of the software on the endocrinologists, a quadric-weighted kappa coefficient was used to determine the agreement between the software and endocrinologists before and after software implementation. Kappa values of 0.01–0.20 indicate none to slight agreement, values of 0.21–0.40 indicate a fair agreement, values of 0.41–0.60 indicate a moderate agreement, values of 0.61–0.80 indicate a substantial agreement, and values of 0.81–1.00 indicate an almost perfect agreement [23]. The monthly percentage of finishing retinal image grading within the allotted time (three days after image examination) and the monthly RDR rate were also compared. All analyses were conducted using R (Version 3.5.3, R Core Team, R Foundation for Statistical Computing, Vienna, Austria).

## 3. Results

### 3.1. Patients and Images

In June 2019, 716 patients underwent fundus examinations before software implementation, and 1432 retinal images were collected. After excluding images considered to be of poor quality by the software, ophthalmologists, and endocrinologists, a total of 981 (68.5%) images were included for analysis. However, only 468 (65.4%) patients had adequate quality of both retinal images. In October 2019, there were 700 patients with 1400 retinal images after software implementation. A total of 503 (71.9%) patients having adequate quality of both retinal images for grading by the software and the endocrinologists were enrolled for analysis. The rate of adequate image quality judged by endocrinologists was 73% ± 9% before Verisee^TM^ implantation and 78% ± 2% after implantation (*p* > 0.05).

### 3.2. Sensitivity, Specificity, and AUC of the Software at the Image Level

**Figure 1:**
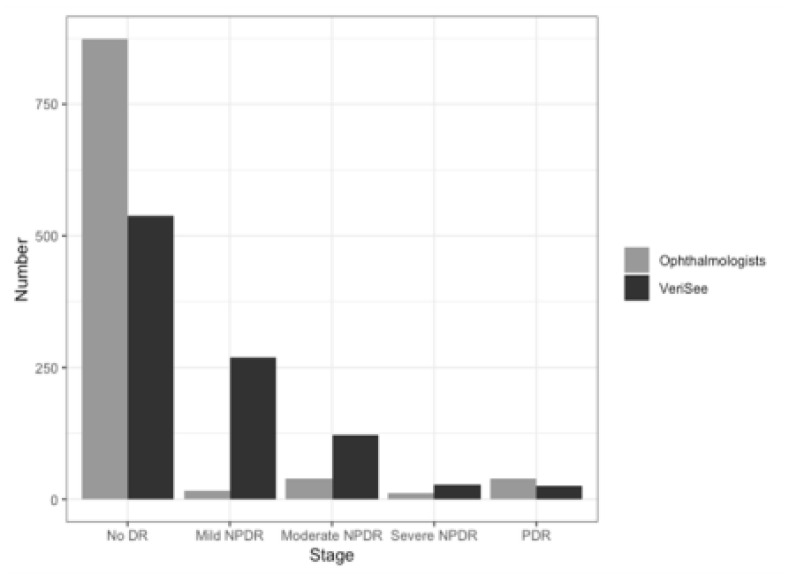
Distribution of diabetic retinopathy severity graded based on VeriSee^TM^ and ophthalmologists.

The ophthalmologists graded 873 (89.0%) images as no DR, 17 (1.7%) images as mild NPDR, 39 (4.0%) images as moderate NPDR, 12 (1.2%) images as severe NPDR, and 40 (4.1%) images as PDR. The software graded 537 (54.7%) images as no DR, 269 (27.4%) images as mild NPDR, 122 (12.4%) images as moderate NPDR, 27 (2.8%) images as severe NPDR, and 26 (2.7%) images as PDR, as shown in Figure 1. A total of 91 (9.3%) and 175 (17.8%) retinal images were graded as RDR by the ophthalmologists and the software, respectively. The sensitivity and specificity of the software to detect RDR were 0.91 (95% CI: 0.83–0.96) and 0.90 (95% CI: 0.87–0.92), respectively, and the AUC was 0.90 (95% CI: 0.87–0.93; Table 1) at the image level. 

### 3.3. Sensitivity, Specificity, and AUC at the Patient Level

**Figure 2:**
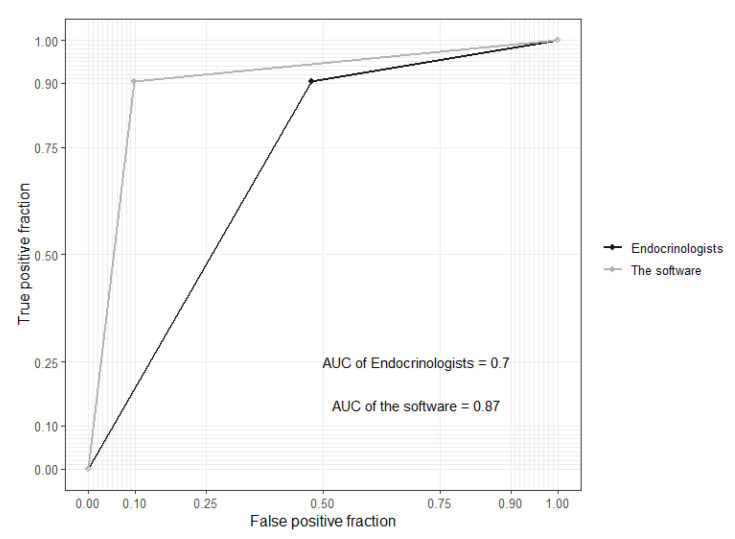
The area under the curve of the receiver operating characteristic curve (AUC) calculated at the patient level for VeriSee^TM^ and the endocrinologists.

Of the 716 patients, 468 (65.4%) were considered adequate quality for grading by the software, the ophthalmologists, and the endocrinologists. The numbers of patients graded as RDR by the software, ophthalmologists, and endocrinologists were 117 (25%), 57 (12.2%), and 258 (55.1%), respectively. As shown in Table 2, the sensitivity, specificity, and AUC for the software to detect RDR were 0.91 (95% CI: 0.81–0.97), 0.84 (95% CI: 0.80–0.87), and 0.87 (95% CI: 0.83–0.91), respectively. For the endocrinologists to detect RDR, the sensitivity, specificity and AUC were 0.91 (95% CI: 0.81–0.97), 0.50 (95% CI: 0.45–0.55), and 0.70 (95% CI: 0.66–0.74), respectively (Figure 2).

### 3.4. Comparison before and after Implementation of the Software

**Figure 3:**
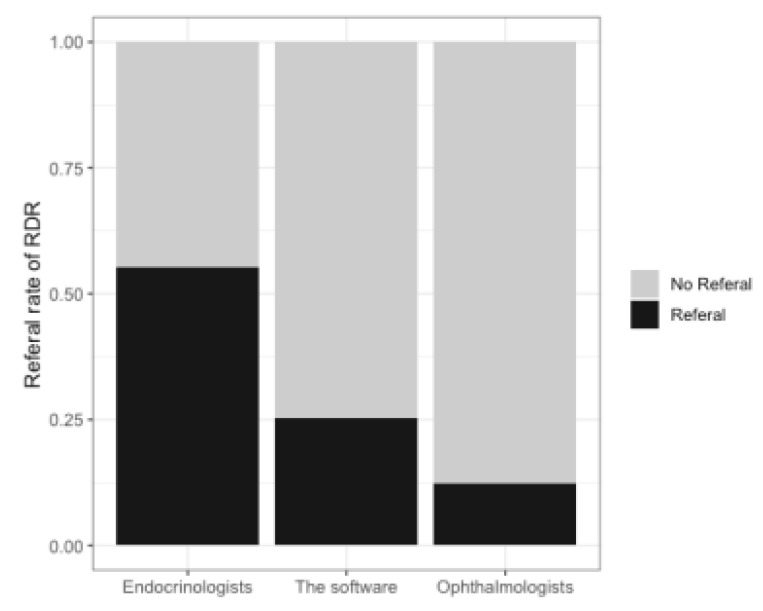
The rate of referable diabetic retinopathy (RDR) graded according to the endocrinologists, VeriSee^TM^, and ophthalmologists.

Before implementation, the monthly RDR rate graded by the endocrinologists was 55.1% (software: 27%; ophthalmologists: 9%, Figure 3). The monthly RDR rate after implementation decreased to 42.9%. The monthly percentage of finishing grading within three days after the examination was 66.8% before implementation, and this increased to 77.6% after implementation (Table 3).

**Figure 4:**
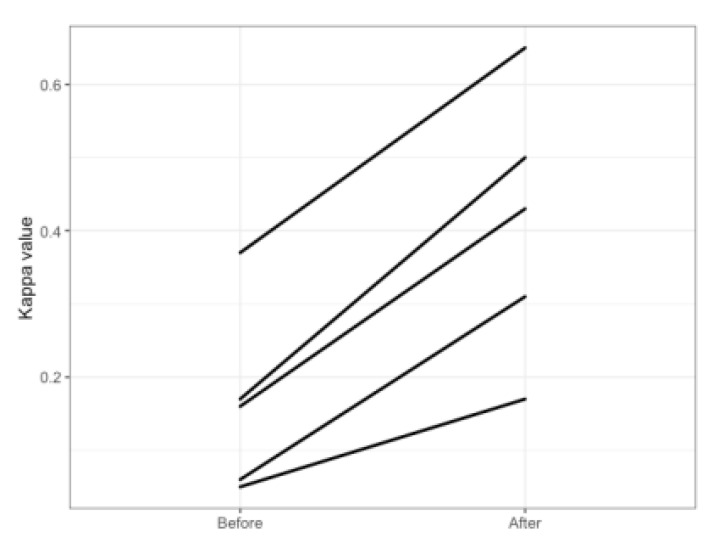
The kappa value for referable diabetic retinopathy (RDR) between each endocrinologist and VeriSee^TM^ before and after software implementation.

The characteristics of each endocrinologist and the kappa values before and after implementation are listed in Table 4. The overall kappa values were low before implementation and increased after implementation of the software. However, there was heterogeneity in the improvement of the kappa values, ranging from 0.17 to 0.65 after implementation (Figure 4). 

## 4. Discussion

In the present study, we evaluated the impact of deploying the deep-learning-based software for RDR diagnosis in clinical practice. A difference in the performance of the software was observed between implementation in laboratory and real-world settings. Therefore, validation with local datasets according to local clinical practice is important before its implementation. Although software implantation was found to have a potential benefit on lessening the workload, clinical physicians’ acceptance of the new technology varied. 

It has been reported that some types of deep-learning-based software demonstrated a high level of performance in the laboratory but reduced sensitivity and specificity in real-world practice [12,24]. Ting et al. [12] externally validated their deep learning algorithm, which showed various levels of specificity from 73.3% to 92.2% for detecting RDR in ten multiethnic clinical datasets, despite there being a specificity of 91.6% in the primary validation dataset. Verbraak et al. [24] reported a deep-learning-based device for RDR with a sensitivity of 79.4% and specificity of 93.8% in the primary care setting, despite the fact that the device had a sensitivity of 87.2% and specificity of 90.7% in the original report [16]. In line with previous reports, our study showed a difference in performance between laboratory and real-world practice. In addition, we found a slight drop in specificity at the patient level compared to that at the image level. Physicians consider referring a patient to an ophthalmologist when one eye reveals RDR, even though the other eye is in good condition. Patient-based judgment is different from how the software is trained based on images. Therefore, validation of the accuracy of the software at both the image and patient levels is warranted before implementation. 

The performance of non-retinal specialists on DR has been well investigated. It was reported that the diagnostic sensitivity and specificity of PDR diagnosis made by physicians other than ophthalmologists were 49% and 84%, respectively, and the rate of correct PDR diagnosis made by endocrinologists was only 31% [25]. Suboptimal sensitivity and specificity should be alerted for non-retinal specialists to detect DR [26]. In the present study, the endocrinologists had a good sensitivity level but a relatively low specificity level regarding the diagnosis of RDR. A possible reason for the high sensitivity with low specificity is that endocrinologists tended to refer a patient if they were uncertain of the diagnosis to avoid the misdiagnosis of patients with RDR. Therefore, the monthly referral rate for RDR was 55% before the implementation of the software, and this was surprisingly higher than the DR prevalence. With the assistance of the software, the monthly rate of RDR graded by endocrinologists was closer to the true prevalence in Taiwan [21,22]. Our study also demonstrated that deep-learning-based software is helpful as it lessens the workload of non-retinal specialists by decreasing the time spent on grading retinal images and increasing the rate of finishing grading within the allotted time.

It is notable that our results showed heterogeneous acceptance among the endocrinologists for diagnosis made by software according to the kappa value. Despite the evidence regarding the high performance of the software [18], not every endocrinologist followed the DR grades assessed using the software in the present study. The human and machine interaction is complex, and further studies are needed to determine the important factors that influence clinicians’ acceptance.

The strength of this study is that we included endocrinologists, who play an important role in DR screening and are the main graders of retinal image in Taiwan. To the best of our knowledge, this is the first report to evaluate the clinical impact of deep-learning-based software on regional graders. However, the present study has several limitations. First, the sample size was relatively small, and we evaluated the impact of the software shortly after its implementation. A long-term investigation with a large sample size is needed in the future. Second, macula edema was not investigated in the study, because the software was not developed for the detection of macula edema. Finally, the percentage of poor quality reached approximately 30% in our retinal images captured using a non-mydriatic fundoscopy, and this percentage is higher than that found in previous reports [27]. Although the use of mydriatic retinal images could improve image quality, the use of non-mydriatic image was more practical due to convenience [28]. 

## 5. Conclusions

We observed a gap between the use of deep-learning-based software in laboratory and real-world settings. We addressed the importance of clinical validation using local datasets for real-world practice. We also demonstrated the potential benefit of deep-learning-based software in terms of decreasing the time spent on grading retinal images. However, the interaction between clinicians and the software appears to be complex. The acceptance of this new technology was found to vary between clinicians, and individual differences in clinical use require further investigation.

## Figures and Tables

**Table 1:** The performance of the software for diagnosing referable diabetic retinopathy (RDR) at the image level.

	VeriSee^TM^
Number of patients	-
Number of images	981
Sensitivity (95% CI)	0.91 (0.83–0.96)
Specificity (95% CI)	0.90 (0.87–0.92)
AUC (95% CI)	0.90 (0.87–0.93)
F1 score	0.62 (0.58–0.65)
Balanced accuracy	0.90 (0.87–0.91)

AUC = area under the receiver operating characteristic curve; CI = confidence interval.

**Table 2:** The performance of software and the endocrinologists for diagnosing referable diabetic retinopathy at the patient level.

	VeriSee^TM^	Endocrinologists
Number of patients	468	468
Sensitivity (95% CI)	0.91 (0.81–0.97)	0.91 (0.81–0.97)
Specificity (95% CI)	0.84 (0.80–0.87)	0.50 (0.45–0.55)
AUC (95% CI)	0.87 (0.83–0.91)	0.70 (0.66–0.74)
F1 score (95% CI)	0.58 (0.54–0.63)	0.33 (0.28–0.37)
Balanced accuracy (95% CI)	0.87 (0.83–0.89)	0.70 (0.65–0.74)

AUC = area under the receiver operating characteristic curve; CI = confidence interval.

**Table 3:** The performance of endocrinologists before and after implementation of the software.

	Before	After
Monthly RDR rate	55.1% (258/468)	42.9% (216/503)
Monthly rate of finishing grading on time *	66.8% (478/716)	77.6% (543/700)

CI = confidence interval, RDR = referable diabetic retinopathy. * Within three days after fundus examination.

**Table 4:** The characteristics and Kappa coefficients of the five endocrinologists.

	Experience * (Years)	Accuracy ^†^	Images ^‡^	Kappa ^§^		
				Before	After	Change
1	2	0.71	209	0.17	0.50	0.33
2	8	0.72	257	0.16	0.43	0.27
3	11	0.7	230	0.06	0.31	0.25
4	13	0.61	189	0.05	0.17	0.12
5	17	0.77	121	0.37	0.65	0.28

* Experience: years of working as endocrinologist; ^†^ Accuracy: the diagnostic accuracy of referable diabetic retinopathy by the endocrinologists, and the grading by the ophthalmologists as the reference standard. ^‡^ Image: the number of images graded by each endocrinologist. ^§^ Kappa coefficient: the agreement between the software and endocrinologists.

## Data Availability

The data presented in this study are available on request from the corresponding author. The data are not publicly available due to privacy.

## Supplementary Materials

The following are available online at https://www.mdpi.com/2075-1729/11/3/200/s1.
