# Supplementary figures and images for: The Clinical Influence after Implementation of Convolutional Neural Network-Based Software for Diabetic Retinopathy Detection in the Primary Care Setting
Source: Life (Basel). 2021 Mar 5;11(3):200. doi: 10.3390/life11030200 (PMC8035657; doi:10.3390/life11030200)

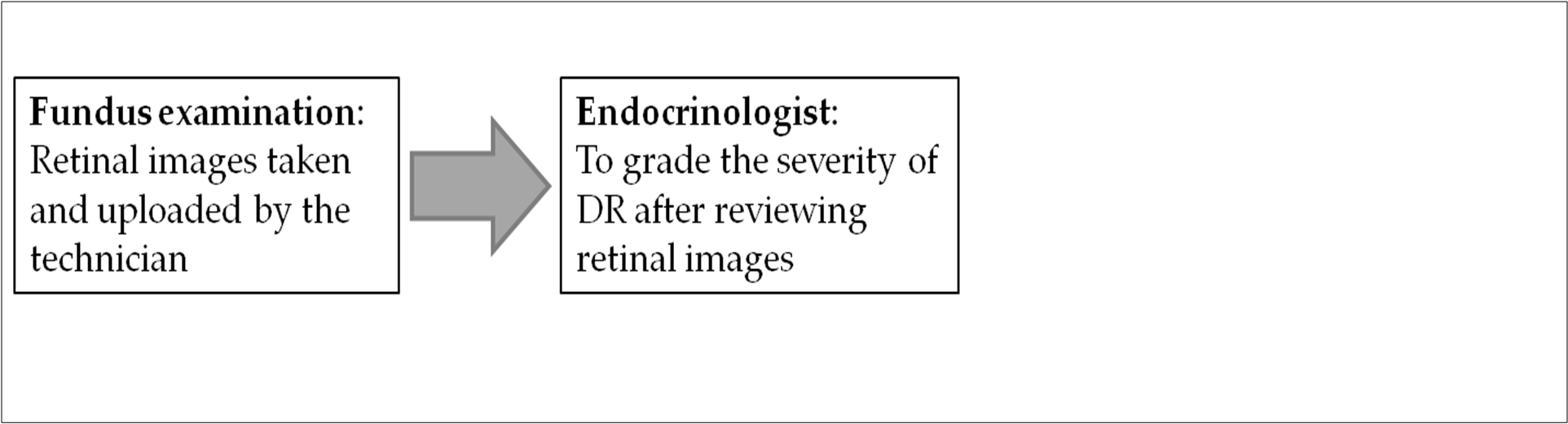

Supplement: Supplementary file 1 [file life-11-00200-s001.zip › Supplement Figure S1.tif]

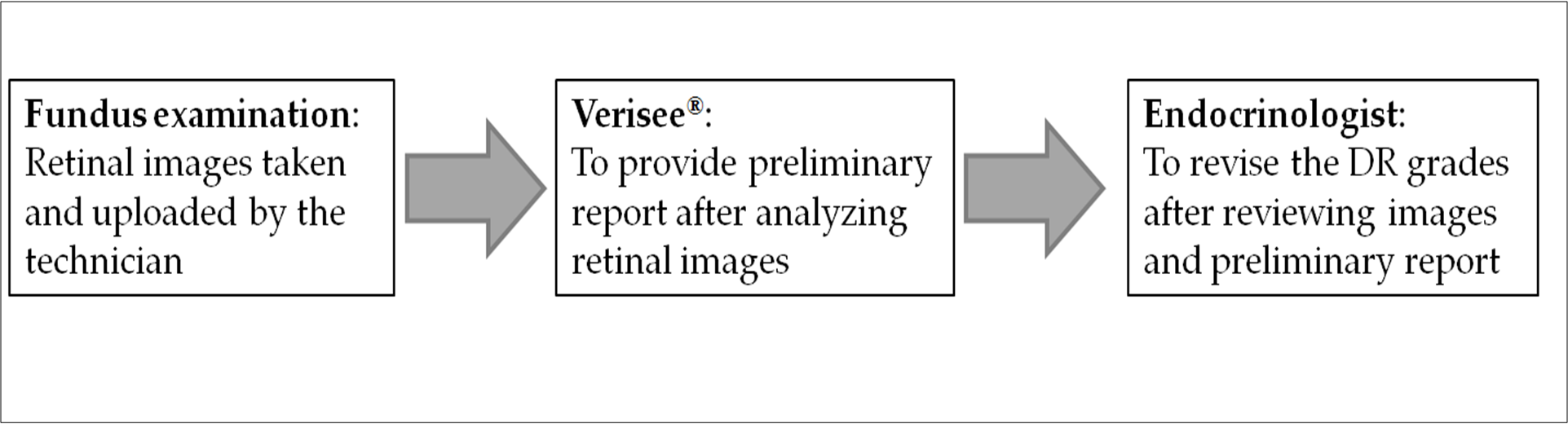

Supplement: Supplementary file 1 [file life-11-00200-s001.zip › Supplement Figure S2.tif]
